# Supplementary material for: Development of a predictive algorithm for patient survival after traumatic injury using a five analyte blood panel
Source: medRxiv. 2024 Jun 11:2024.04.22.24306188. Preprint. [Version 2] doi: 10.1101/2024.04.22.24306188 (PMC11188118; doi:10.1101/2024.04.22.24306188)
Supplement: 1 [file NIHPP2024.04.22.24306188V2-supplement-1.pdf]

medRxiv preprint doi: https://doi.org/10.1101/2024.04.22.24306188; this version posted June 11, 2024. The copyright holder for this preprint (which was not certified by peer review) is the author/funder, who has granted medRxiv a license to display the preprint in perpetuity. This article is a US Government work. It is not subject to copyright under 17 USC 105 and is also made available for use under a CC0 license.

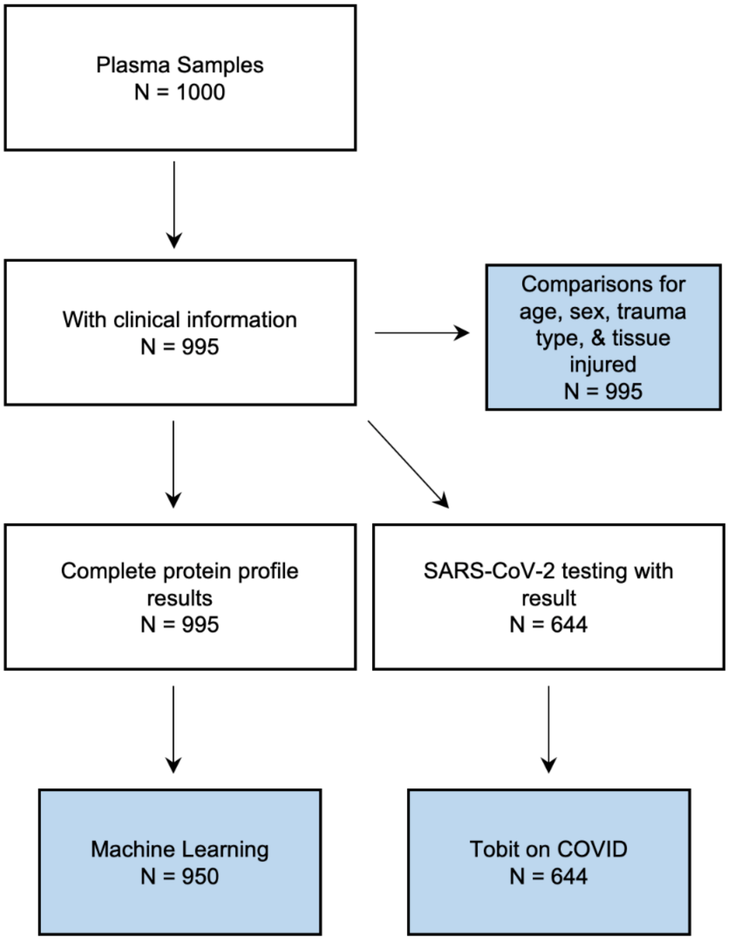

Supplemental Figure 1 | Sample and data analysis workflow. White boxes = sample input. Blue boxes = analyses.

medRxiv preprint doi: https://doi.org/10.1101/2024.04.22.24306188; this version posted June 11, 2024. The copyright holder for this preprint (which was not certified by peer review) is the author/funder, who has granted medRxiv a license to display the preprint in perpetuity.
This article is a US Government work. It is not subject to copyright under 17 USC 105 and is also made available for use under a CC0 license.

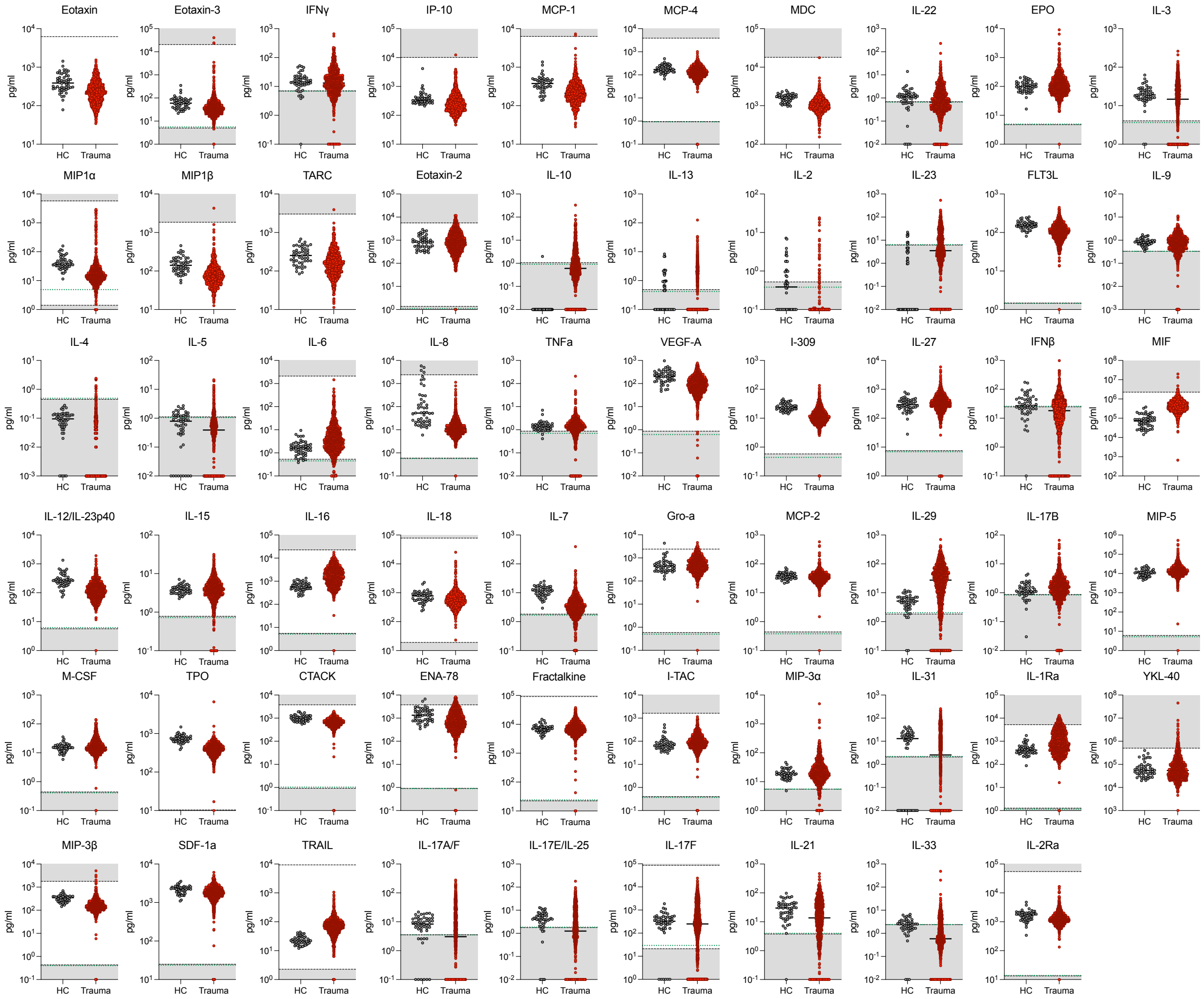

Supplemental Figure 2 | Raw data display for all analytes measured in full cohort. Grey = Healthy controls (HC); Red Points = Trauma Patients. Green lline = threshold for healthy control samples, Black line = threshold for trauma samples. Grey shaded areas = above or below highest known standard curve value.

medRxiv preprint doi: https://doi.org/10.1101/2024.04.22.24306188; this version posted June 11, 2024. The copyright holder for this preprint (which was not certified by peer review) is the author/funder, who has granted medRxiv a license to display the preprint in perpetuity. This article is a US Government work. It is not subject to copyright under 17 USC 105 and is also made available for use under a CC0 license.

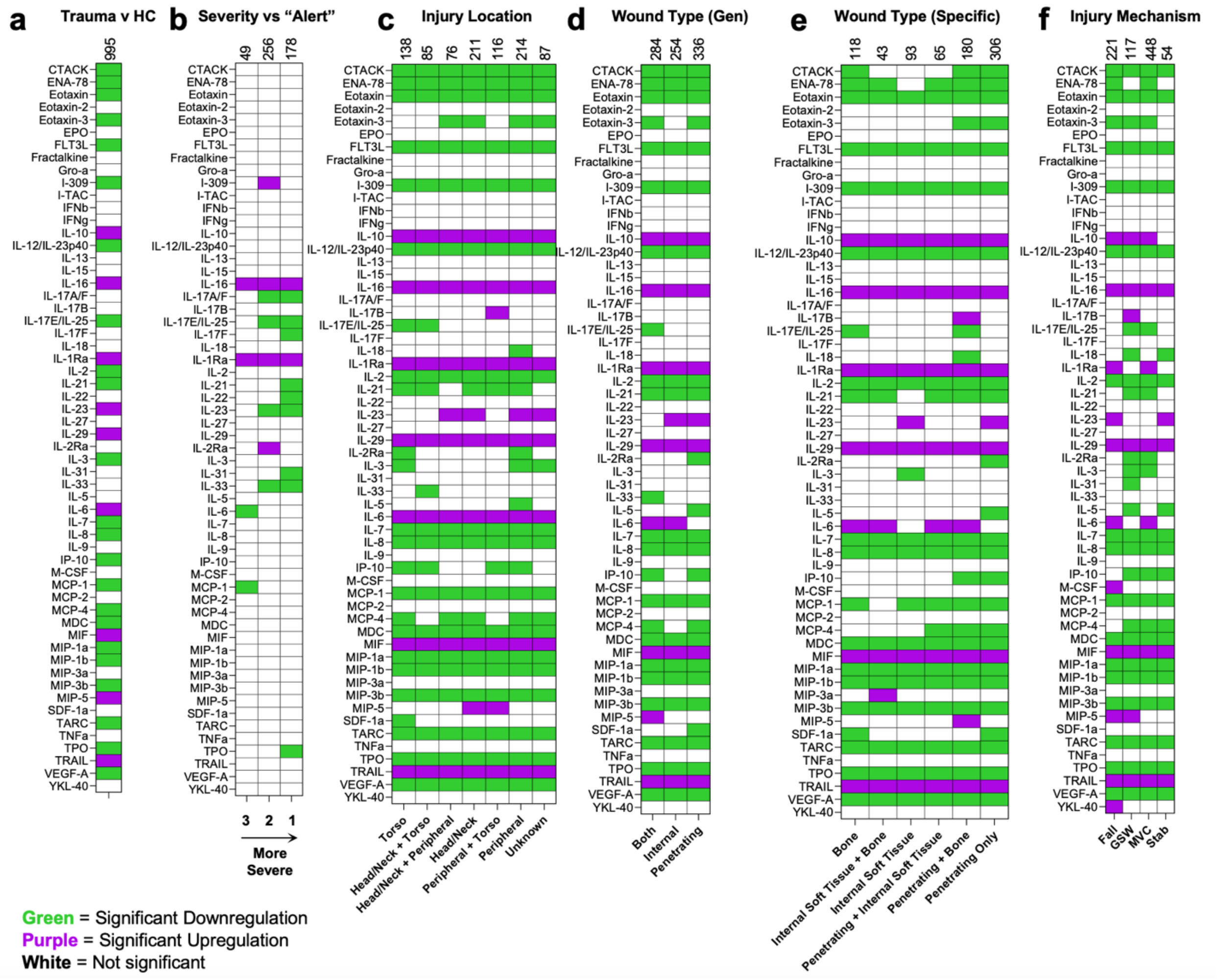

Supplemental Figure 3 | Binary Heatmaps of Significant Tobit Estimates. Heatmaps of statistically significant Tobit estimates for (a) Trauma versus healthy controls, (b) Different trauma levels compared to trauma level 1, (c) Injury location, (d) general wound type, (e) Specific wound type, and (f) injury mechanism.

medRxiv preprint doi: https://doi.org/10.1101/2024.04.22.24306188; this version posted June 11, 2024. The copyright holder for this preprint (which was not certified by peer review) is the author/funder, who has granted medRxiv a license to display the preprint in perpetuity. This article is a US Government work. It is not subject to copyright under 17 USC 105 and is also made available for use under a CC0 license.

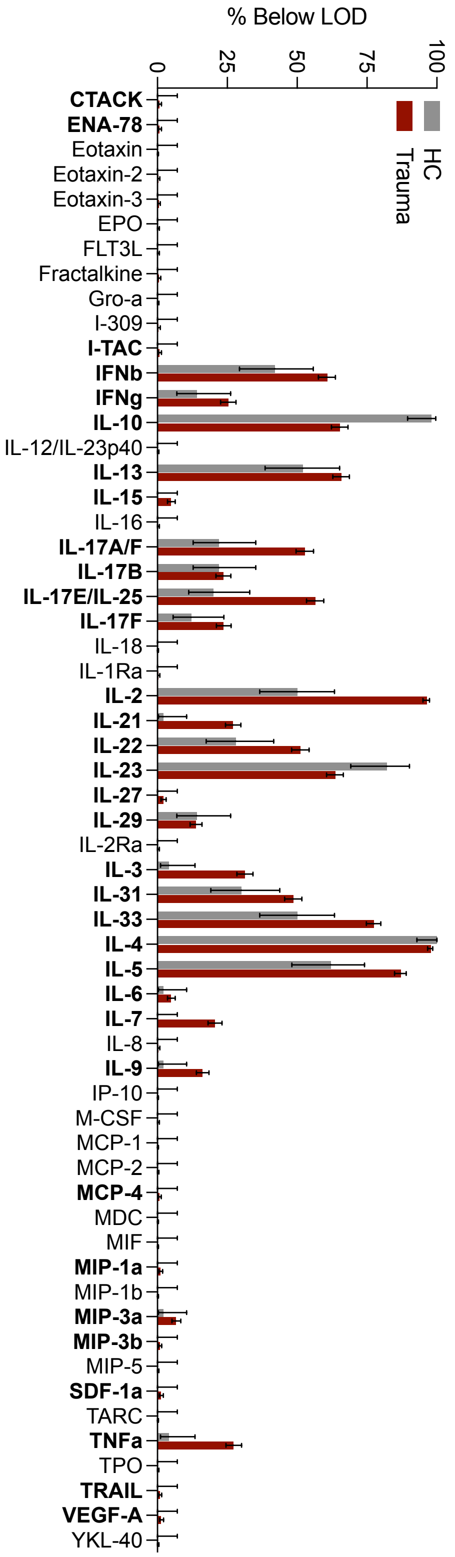

Supplemental Figure 4 | Proportion below limit of detection. Proportion of samples that were below the limit of detection (LOD) for the assay. Grey = Healthy controls, Red = trauma patients. Data are % below LOD ± 95% confidence intervals (Wilson).

medRxiv preprint doi: https://doi.org/10.1101/2024.04.22.24306188; this version posted June 11, 2024. The copyright holder for this preprint (which was not certified by peer review) is the author/funder, who has granted medRxiv a license to display the preprint in perpetuity.
This article is a US Government work. It is not subject to copyright under 17 USC 105 and is also made available for use under a CC0 license.

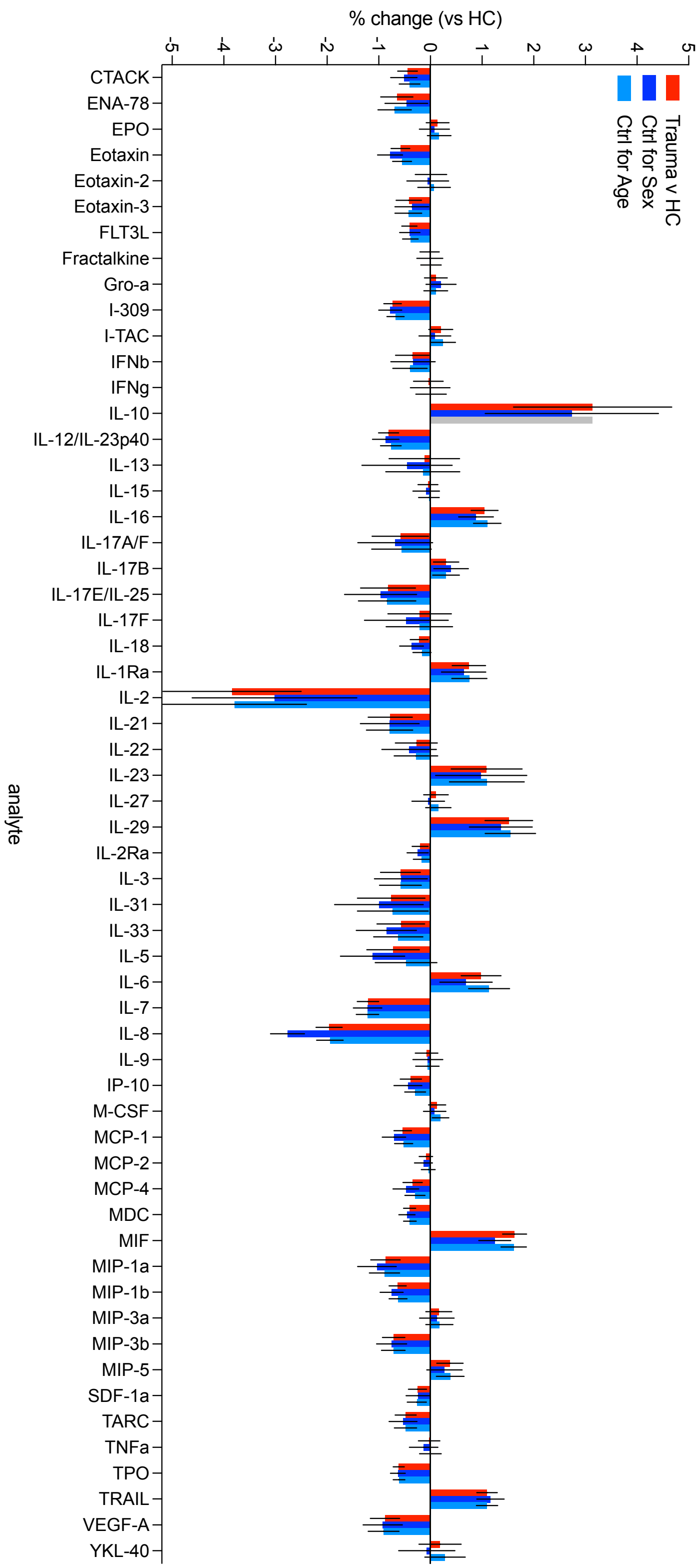

Supplemental Figure 5 | Estimates of change from healthy volunteers when controlled for sex and age. Tobit estimates for trauma vs healthy controls (HC) with overall (red), controlled for sex (blue), and controlled for age (light blue). Data are log change ± 95% confidence intervals.

medRxiv preprint doi: https://doi.org/10.1101/2024.04.22.24306188; this version posted June 11, 2024. The copyright holder for this preprint (which was not certified by peer review) is the author/funder, who has granted medRxiv a license to display the preprint in perpetuity. This article is a US Government work. It is not subject to copyright under 17 USC 105 and is also made available for use under a CC0 license.

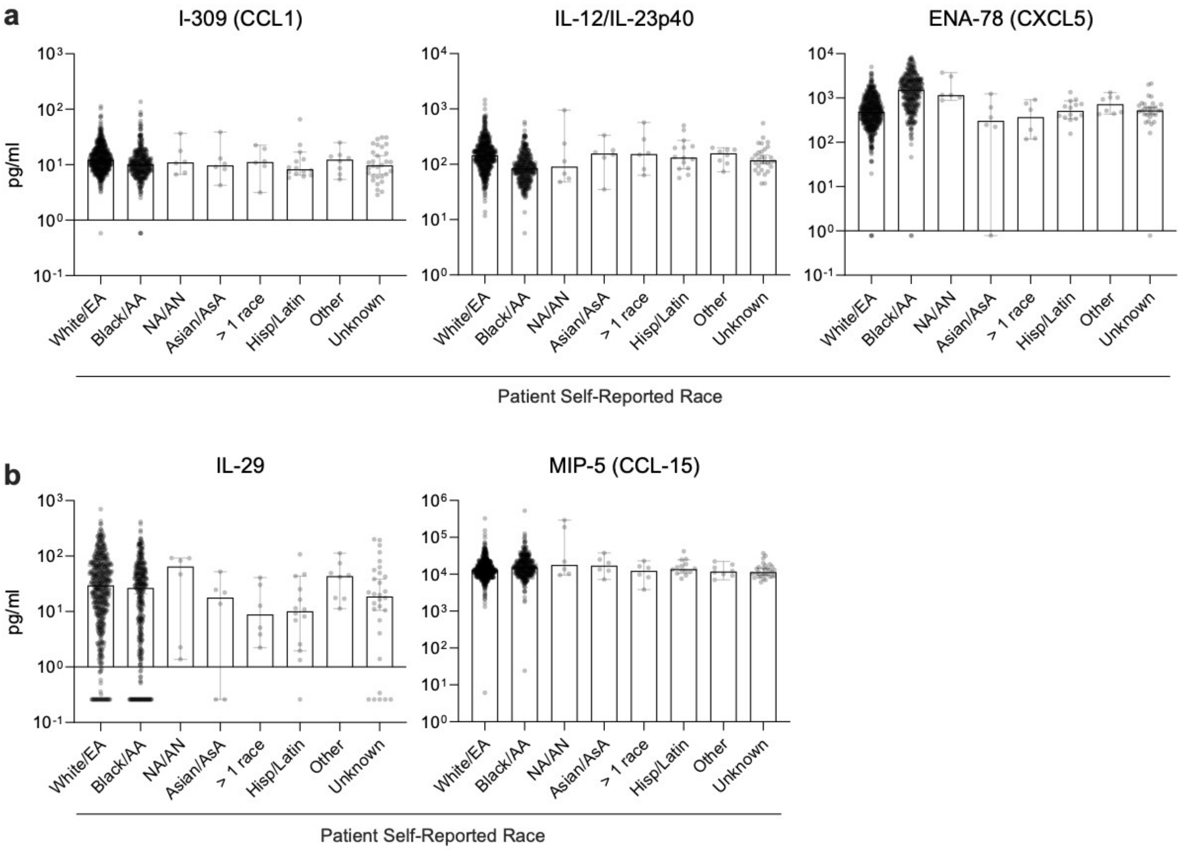

Supplemental Figure 6 | Effect of self-reported race on concentration of cytokines and chemokines in the blood of trauma patients. (a) Novel downregulated proteins as a function of self-reported race. (b) Novel upregulated proteins as a function of self-reported race. EA = European American, AA = African American, NA/AN = Native American/Alaska Native, AsA = Asian American. Data are mean ± standard deviation.

medRxiv preprint doi: https://doi.org/10.1101/2024.04.22.24306188; this version posted June 11, 2024. The copyright holder for this preprint (which was not certified by peer review) is the author/funder, who has granted medRxiv a license to display the preprint in perpetuity.
This article is a US Government work. It is not subject to copyright under 17 USC 105 and is also made available for use under a CC0 license.

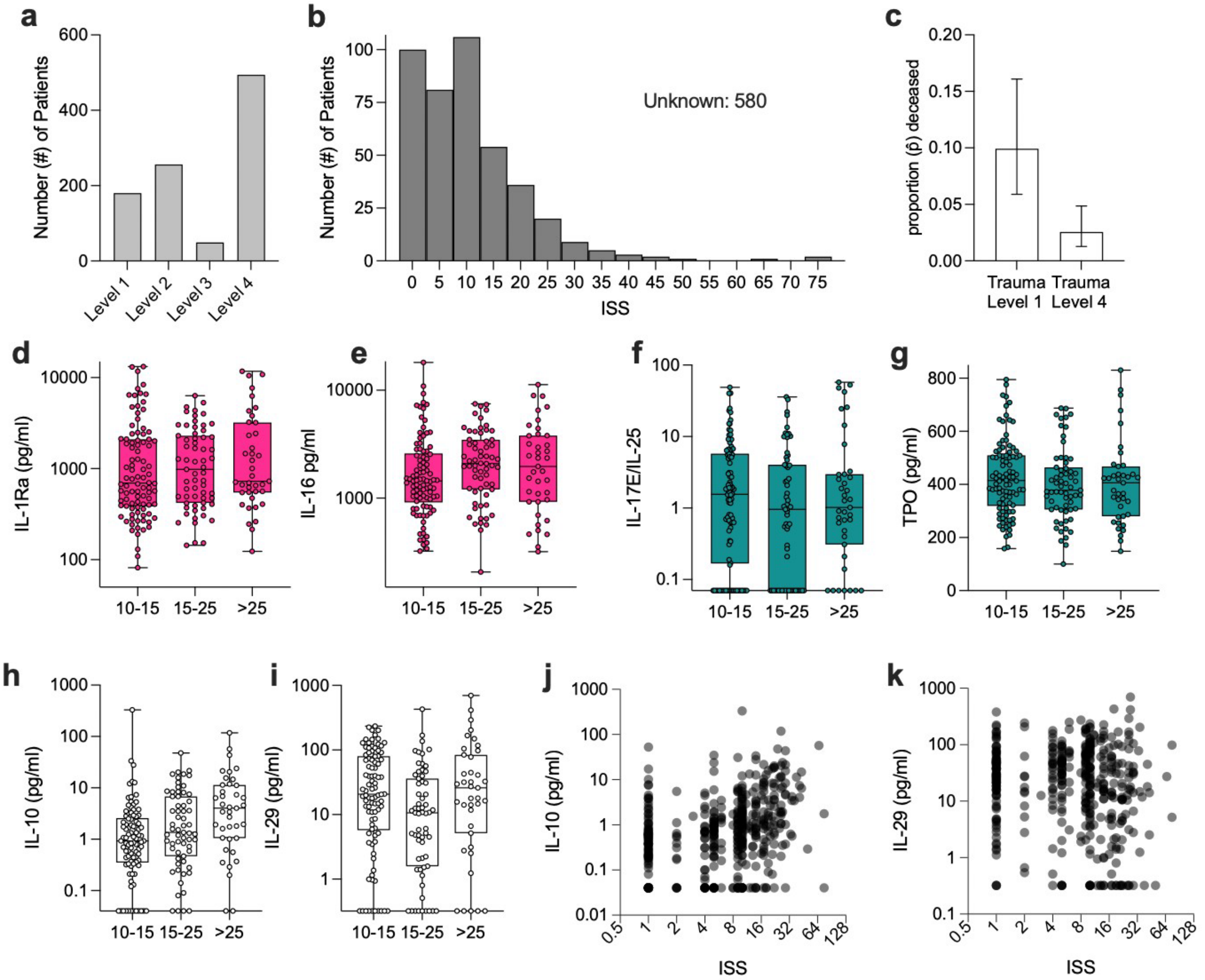

Supplemental Figure 7 | Trauma level correlates with risk of death and protein concentration patterns are conserved with those that have an injury severity score (ISS). (a) Number of patients categorized into different trauma levels (Level 1 = most severe trauma center activation, Level 4 = least severe trauma center activation). (b) Distribution of ISS including those listed with ISS (including those with one injury). (c) Probability of death when admitted under trauma activation level 1 versus trauma activation level 4. (d – g) Distribution of cytokine/chemokine concentration of upregulated (d, e) IL-1Ra and IL-16 respectively, and downregulated (f, g) IL-17E/IL-25 and TPO respectively, proteins as determined by Level 1 versus Level 4 concentration. (h, i) Concentration of (h) IL-10 and (i) IL-29 by ISS level and (j-k) Correlation of (j) IL-10 and (k) IL-29 concentrations as a function of ISS.

medRxiv preprint doi: https://doi.org/10.1101/2024.04.22.24306188; this version posted June 11, 2024. The copyright holder for this preprint (which was not certified by peer review) is the author/funder, who has granted medRxiv a license to display the preprint in perpetuity. This article is a US Government work. It is not subject to copyright under 17 USC 105 and is also made available for use under a CC0 license.

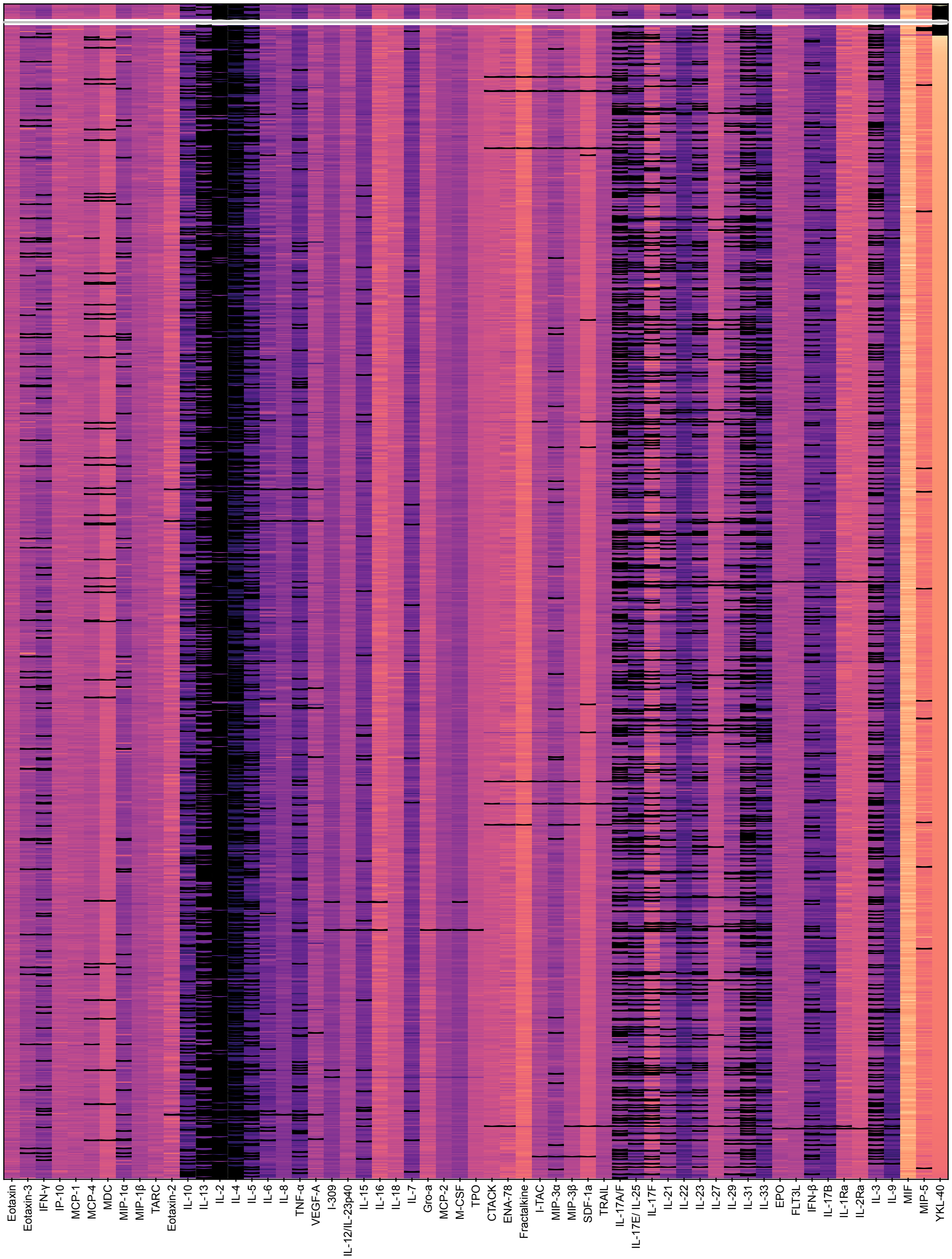

Supplemental Figure 8 | Phenotypes of sub-cluster of patients with variable cytokine/chemokine levels. Log-normalized concentration (pg/ml), black = above or below limit of detection (LOD).

medRxiv preprint doi: https://doi.org/10.1101/2024.04.22.24306188; this version posted June 11, 2024. The copyright holder for this preprint (which was not certified by peer review) is the author/funder, who has granted medRxiv a license to display the preprint in perpetuity.
This article is a US Government work. It is not subject to copyright under 17 USC 105 and is also made available for use under a CC0 license.

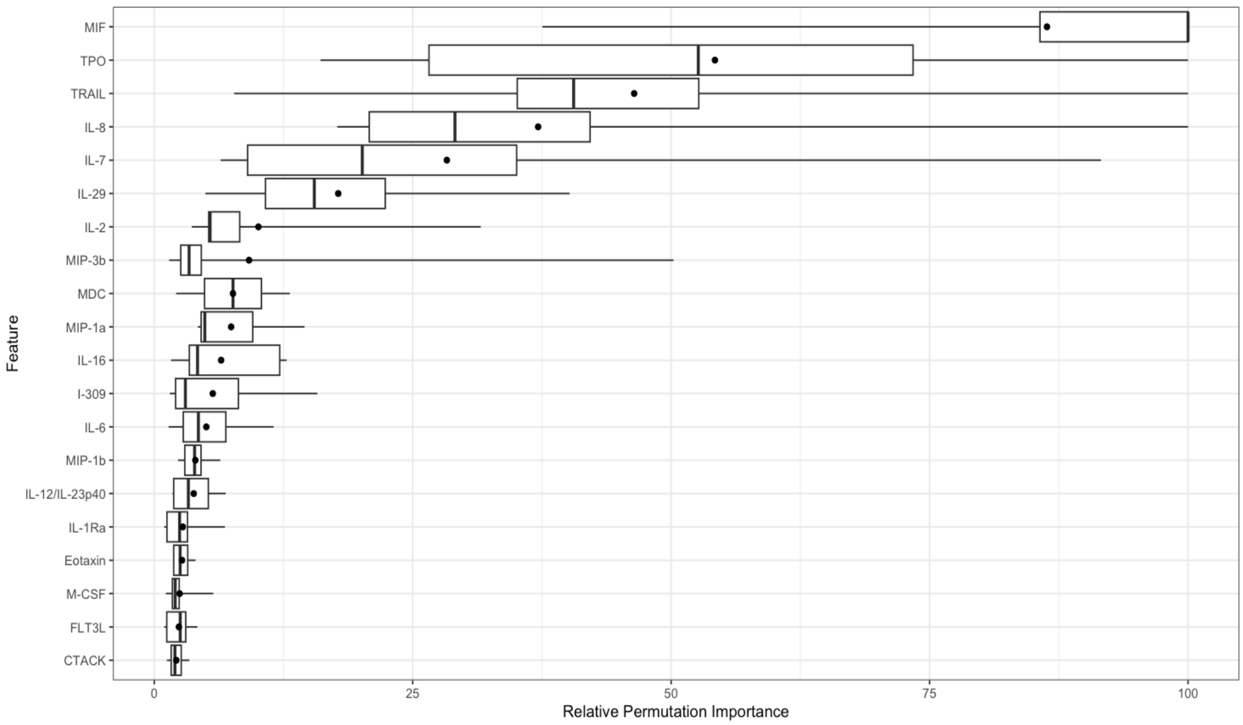

Supplemental Figure 9 | Relative permutation importance of different proteins on identification of trauma versus healthy control via machine learning. Boxplots are the protein's minimum, first quartile, median, mean (black dot), third quartile, and maximum relative importance values across 10 model iterations. Importance is ordered by average relative importance across all iterations.

medRxiv preprint doi: https://doi.org/10.1101/2024.04.22.24306188; this version posted June 11, 2024. The copyright holder for this preprint (which was not certified by peer review) is the author/funder, who has granted medRxiv a license to display the preprint in perpetuity. This article is a US Government work. It is not subject to copyright under 17 USC 105 and is also made available for use under a CC0 license.

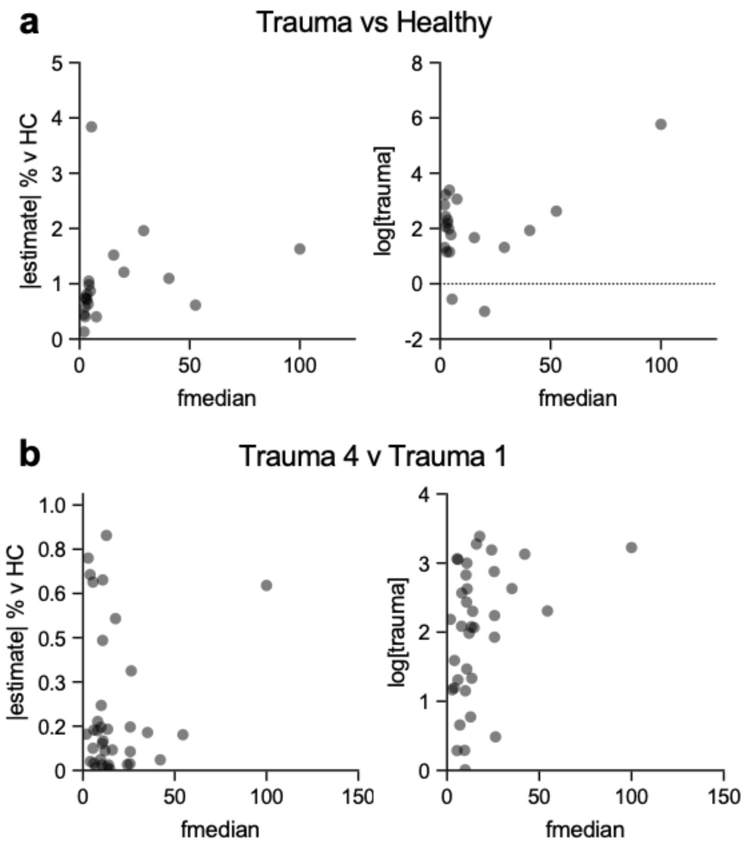

Supplemental Figure 10 | Association of median relative importance with absolute percent change and analyte concentration. (a) Median relative importance (fmedian) of proteins across 10 model iterations versus its absolute percent change of concentration in trauma patients versus healthy controls (left) and concentration of analyte (right) in comparison between healthy controls and trauma. (b) Median relative importance (fmedian) of proteins across 10 model iterations versus its absolute percent change of concentration from trauma level 1 to trauma level 4 (left) and concentration of analyte (right) in comparison between trauma level.

medRxiv preprint doi: https://doi.org/10.1101/2024.04.22.24306188; this version posted June 11, 2024. The copyright holder for this preprint (which was not certified by peer review) is the author/funder, who has granted medRxiv a license to display the preprint in perpetuity.
This article is a US Government work. It is not subject to copyright under 17 USC 105 and is also made available for use under a CC0 license.

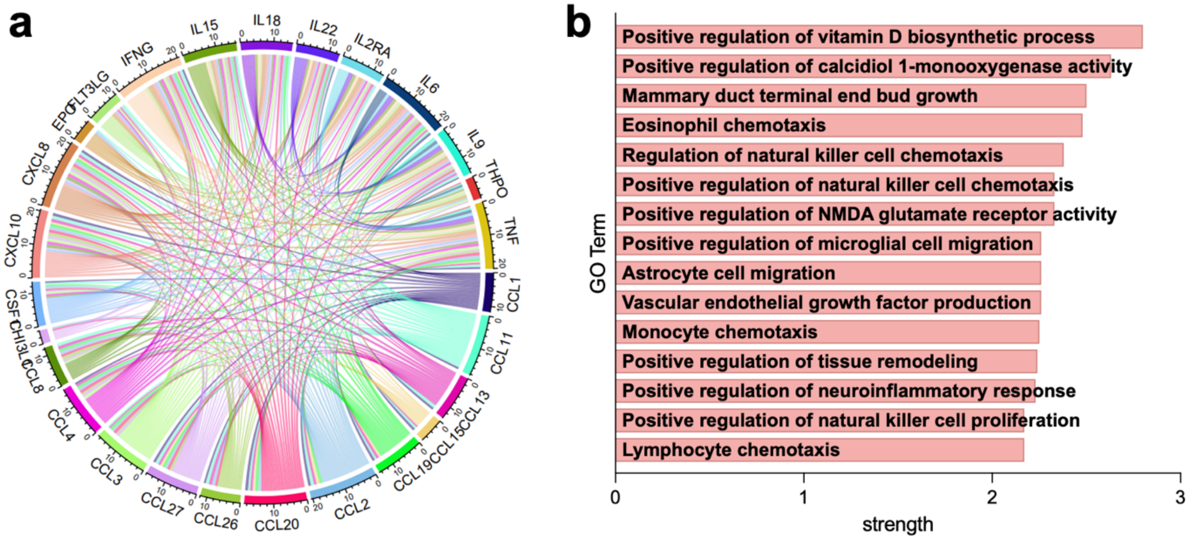

Supplemental Figure 11 | STRING analysis of proteins upregulated with age. (a) Chord diagram generated based on STRING analysis of interactions among proteins upregulated with age. (b) STRING database gene ontology enrichment of biological processes from proteins upregulated with age (top 15 in strength of enrichment, FDR < 0. 05).

medRxiv preprint doi: https://doi.org/10.1101/2024.04.22.24306188; this version posted June 11, 2024. The copyright holder for this preprint (which was not certified by peer review) is the author/funder, who has granted medRxiv a license to display the preprint in perpetuity.
This article is a US Government work. It is not subject to copyright under 17 USC 105 and is also made available for use under a CC0 license.

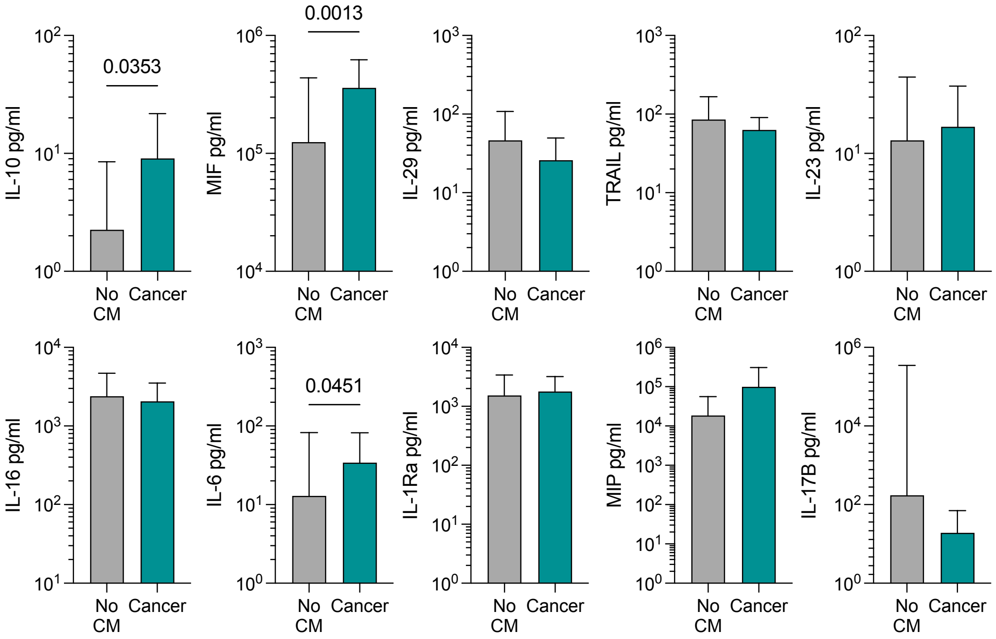

Supplemental Figure 12 | Exploratory analyses of trauma patients with no comorbidities versus those with reported cancer incidence. Grey = no comorbidities (CM), Teal = cancer patients (cancer). Data are mean ± standard deviation, p = student's T test (without correction for multiple comparisons).

medRxiv preprint doi: https://doi.org/10.1101/2024.04.22.24306188; this version posted June 11, 2024. The copyright holder for this preprint (which was not certified by peer review) is the author/funder, who has granted medRxiv a license to display the preprint in perpetuity. This article is a US Government work. It is not subject to copyright under 17 USC 105 and is also made available for use under a CC0 license.

	Count	%
**Location**		
Worcester	154	15.45
Charlotte	219	21.97
Baltimore	208	20.86
Jacksonville	223	22.37
Miami	193	19.36
**Sex**		
Male	725	72.50
Female	272	27.20
Unknown	3	0.30
**Race**		
White or EA	552	55.20
Black or AA	375	37.50
Asian or AsA	6	0.60
Native American/Alaska Native	6	0.60
Other	28	2.80
Unknown	33	3.30
**Ethnicity**		
Hispanic/Latino	162	16.20
Not H/L	818	81.80
Unknown	20	2.00
**Age**		
18 - 39	499	50.51
40 - 70	373	37.75
> 70	113	11.44
Unknown	3	0.30
**Mechanism of Injury**		
MVC	448	37.87
Fall	221	18.68
GSW	117	9.89
Assault	221	18.68
Stab	54	4.56
Fire/burn	17	1.44
Nature/environment	3	0.25
Non-Motorized Transport	4	0.34
Drowning	1	0.08
Other Motorized Transport	16	1.35
Other, specified	77	6.51
Other, unspecified	1	0.08
Unknown	3	0.25
**Tested for COVID?**		
Yes	652	65.20
No	332	33.20
Unknown/NA	16	1.60

Supplemental Table 1 | Demographics and basic trauma information for patients. Sex = biologic sex. Race = Self-reported race (EA = European American, AA = African American, AsA = Asian American). Ethnicity = Self-reported ethnicity (H/L = Hispanic/Latino). MVC = motor vehicle crash, GSW = gunshot wound or shotgun wound.

medRxiv preprint doi: https://doi.org/10.1101/2024.04.22.24306188; this version posted June 11, 2024. The copyright holder for this preprint (which was not certified by peer review) is the author/funder, who has granted medRxiv a license to display the preprint in perpetuity.
This article is a US Government work. It is not subject to copyright under 17 USC 105 and is also made available for use under a CC0 license.

Analyte	Aliases	Reported in humans systemically?	Trauma/Surgery/ Sepsis/TBI/Model Organism?	Reported Trend	This Study	References (PMID)	Notes
CTACK	CCL27	No	In vitro	None reported	DOWN	-	No literature on traumatic injury, only cell culture
ENA-78	CXCL5	No	Surgery	Increased (local tissue)	DOWN	8961388	Hepatectomy
Eotaxin	CCL11	Yes	Surgery	Decreased with trauma	DOWN	24602333	Muscle surgical trauma
Eotaxin-3	CCL26	No	Trauma	Increased (local tissue)	DOWN	29764285	Subdural hematoma fluids
FLT3L		No	TBI	Increased (local tissue)	DOWN	31017044, 25672780	TBI, Protective in MODS
I-309	CCL1	No	TBI	Increased (local tissue)	DOWN	23788036	Neural tissue, mouse
I-TAC	CXCL11	No	TBI	Increased (local tissue)	DOWN	33189916	TBI
IFNb		No	Trauma	Increased (local tissue)	DOWN	27438769	Skin tissue
IFNg		No	Trauma	Decreased (isolated cells)	DOWN	20100328	T cells isolated from trauma patients
IL-10		Yes	Trauma	Increased with trauma	UP	19317866, 11030163, 18092384, 10580806	Multiple trauma types & severities
IL-12/IL-23p40		No		None reported	DOWN	-	No literature on traumatic injury
IL-13		Yes	TBI	No change (plasma)	DOWN	11089904	TBI
IL-15		Yes	Sepsis	Decreased (plasma)	DOWN	36915009	Sepsis in blunt trauma patients
IL-16		Yes	Trauma	Increased (plasma)	UP	15706184	General trauma patients
IL-17A/F		No	OA (Mouse)	Increased (local tissue)	DOWN	32955487	PTOA, mouse model
IL-17B		No		None reported	DOWN	-	No literature on traumatic injury, only autoimmune
IL-17E/IL-25	IL25	No		None reported	DOWN	-	No literature on traumatic injury, only autoimmune
IL-17F		No	OA (Mouse)	Increased (local tissue)	DOWN	32955487	PTOA, mouse model
IL-1Ra		Yes	Trauma	Increased (plasma, PBMCs)	UP	7474003, 7606873	Surgical and traumatic injury
IL-2		Yes	Trauma	Decreased (plasma)	DOWN	3877497, 2956432	Traumatic injury
IL-21		Yes	Trauma	Decreased (plasma)	DOWN	33748428	Down in severe trauma
IL-22		Yes	Trauma	Decreased (plasma)	UP	33748428	Down in severe trauma
IL-23		Yes	Trauma	Decreased (plasma)	UP	33748428	Down in severe trauma
IL-27		Yes	Sepsis	Increased (local tissue, serum)	DOWN	23842867, 31507598	BAL of lung injury, serum of sepsis patients
IL-29	IFNL1	No		None reported	UP	-	No literature on traumatic injury, only autoimmune
IL-3		Yes	Trauma	Decreased (plasma)	DOWN	9126191	Trauma hemorrhage
IL-31		No		None reported	DOWN	-	No literature on traumatic injury, only itch
IL-33		Yes	Trauma	Increased (plasma)	DOWN	33771098	Early in crtically injured patients
IL-4		Yes	Trauma	Decreased (serum)	DOWN	24402554	Orthopedic trauma
IL-5		No		None reported	DOWN	-	No literature on traumatic injury
IL-6		Yes	Trauma	Increased (serum)	UP	24402554	Orthopedic trauma
IL-7		Yes	Trauma	Decreased (serum)	DOWN	31246919	Chest wound + TBI versus TBI alone
IL-8	CXCL8	Yes	Trauma	Conflicting data (serum)	DOWN	24402554, 32837068	Increased in orthopedic trauma, decreased in thoracic trauma
IL-9		Yes	Trauma	Decreased (plasma)	DOWN	33748428	Decreased in severe blunt trauma patients
IP-10	CXCL10	Yes	Trauma	Increased (plasma)	DOWN	19717036	Increased in multi organ failure patients versus other trauma patients
MCP-1	CCL2	Yes	Trauma	Increased (serum)	DOWN	37562231	Greater in nonsurvivors of trauma
MCP-4	CCL13	Yes	TBI	Increased (plasma)	DOWN	29685283	Brain injury (concussed)
MDC	CCL22	No	OA (Rat)	Increased (serum)	DOWN	30623804	Rat model of OA
MIF		Yes	Trauma	Increased (serum)	UP	20499270	Higher in post-trauma MODS
MIP-1a	CCL3	No	Surgery	Increased (local tissue)	DOWN	26543677, 21371163	Increased in wound fluid of surgical wounds
MIP-1b	CCL4	No	Surgery	Increased (local tissue)	DOWN	26543677, 21371163	Increased in wound fluid of surgical wounds
MIP-3a	CCL20, LARC	No		None reported	DOWN	-	No literature on traumatic injury
MIP-3b	CCL19, ELC	No		None reported	DOWN	-	No literature on traumatic injury, only autoimmune
MIP-5	CCL15	No		None reported	UP	-	No literature on traumatic injury
SDF-1a		No	Trauma	Increased (local tissue)	DOWN	28272352	Produced at the site of injury
TARC		Yes	Trauma	Increased (plasma)	DOWN	37120600	Trauma hemorrhagic shock
TNFa		Yes	Trauma	No change (plasma)	DOWN	8230332	General trauma patients
TPO	THPO, MGDF	Yes	Sepsis	Increased (serum)	DOWN	10997881	Sepsis
TRAIL	CD253, TNFSF10	No	Trauma	Increased (local tissue)	UP	21760914	Gene expression, local tissue of elderly bone fractures
VEGF-A	VEGF	Yes	Trauma	Increased (serum)	DOWN	9711425	Polytrauma and burns

Supplemental Table 2 | Literature review of cytokines analyzed and their reported trends in trauma and associated conditions. Review on both GoogleScholar and PubMed search engines using key words “trauma”. “injury”, “wound”, “traumatic injury”, with or without “human”. Publications are only listed that are indexed in PubMed.

medRxiv preprint doi: https://doi.org/10.1101/2024.04.22.24306188; this version posted June 11, 2024. The copyright holder for this preprint (which was not certified by peer review) is the author/funder, who has granted medRxiv a license to display the preprint in perpetuity. This article is a US Government work. It is not subject to copyright under 17 USC 105 and is also made available for use under a CC0 license.

Analyte	Aliases	Reported Trend with Age	This Study	References (PMID)	Notes
CTACK	CCL27	None reported	UP	-	
ENA-78	CXCL5	None reported	DOWN	-	
EPO		Increase with age	UP	25915923	
Eotaxin	CCL11	Increase with age	UP	26080062	
Eotaxin-3	CCL26	Decrease with age	UP	30685456	Dermatitis patients
FLT3L		Increase with age	UP	26080062	
Gro-a	CXCL1	None reported	DOWN	-	
I-309	CCL1	None reported	UP	-	
IFNg		Increase with age	UP		In children
IL-12/IL-23p40		Increase with age	UP	10671301	Evaluated total IL-12
IL-15		Increase with age	UP	16192677	
IL-16		None reported	DOWN	-	
IL-18		Increase with age	UP	21571262	Secreted from dendritic cells (not total)
IL-22		None reported	UP	-	
IL-27		Trended increase	UP		
IL-2Ra	CD25	None reported	UP	-	
IL-6		Debated	UP	1453878, 11213271	Some reports of increase, some decrease with age
IL-8	CXCL8	None reported	UP	-	
IL-9		None reported	UP	-	
IP-10	CXCL10	Increase with age	UP	26080062	Trauma Patients
M-CSF	CSF1	Trended increase	UP	26080062	
MCP-1	CCL2	Increase with age	UP	23039889	Study incorrectly calls MCP-1 as CCL1, cited here under MCP-1
MCP-2	CCL8	None reported	UP	-	
MCP-4	CCL13	Increase with age	UP	26080062	
MIP-1a	CCL3	None reported	UP	-	
MIP-1b	CCL4	Decrease with age	UP	30448299	Trauma Patients
MIP-3a	CCL20, LARC	None reported	UP	-	
MIP-3b	CCL19, ELC	None reported	UP	-	
MIP-5	CCL15	None reported	UP	-	
TNFa		Increase with age	UP	10931139	
TPO	THPO, MGDF	None reported	UP	-	
TRAIL	CD253, TNFSF10	None reported	DOWN	-	
VEGF-A	VEGF	Increase with age	UP	26080062	
YKL-40	CHI3L1	Increase with age	UP	18070151	

Supplemental Table 3 | Literature review of cytokines analyzed and their reported trends with age in human subjects. Review on both GoogleScholar and PubMed search engines using key words “age”, “increase”, “decrease”, “correlate with age”, and “human” or “patient”. Publications are only listed that are indexed in PubMed.

medRxiv preprint doi: https://doi.org/10.1101/2024.04.22.24306188; this version posted June 11, 2024. The copyright holder for this preprint (which was not certified by peer review) is the author/funder, who has granted medRxiv a license to display the preprint in perpetuity. This article is a US Government work. It is not subject to copyright under 17 USC 105 and is also made available for use under a CC0 license.

Analyte	ALIAS	Description in STRING
CTACK	CCL27	C-C motif chemokine 27; Chemotactic factor that attracts skin-associated memory T- lymphocytes. May play a role in mediating homing of lymphocytes to cutaneous sites. Binds to CCR10. [a.k.a. R-HSA-388396, IL-11 R-alpha-locus chemokine, R-HSA-162582, CTACK]
ENA-78	CXCL5	C-X-C motif chemokine 5; Involved in neutrophil activation. In vitro, ENA-78(8-78) and ENA-78(9-78) show a threefold higher chemotactic activity for neutrophil granulocytes; Belongs to the intercrine alpha (chemokine CxC) family. [a.k.a. R-HSA-388396, CAC42884.1, chemokine (C-X-C motif) ligand
Eotaxin	CCL11	In response to the presence of allergens, this protein directly promotes the accumulation of eosinophils, a prominent feature of allergic inflammatory reactions. Binds to CCR3. [a.k.a. eotaxin-1, R-HSA-162582, ENST00000305869, eotaxin, Eotaxin, ...]
Eotaxin-2		C-C motif chemokine 24; Chemotactic for resting T-lymphocytes, and eosinophils. Has lower chemotactic activity for neutrophils but none for monocytes and activated lymphocytes. Is a strong suppressor of colony formation by a multipotential hematopoietic progenitor cell line. Binds to CCR3. [a.k.a.
Eotaxin-3	CCL26	C-C motif chemokine 26; Chemoattractant for eosinophils and basophils. Acts as a ligand for C-C chemokine receptor CCR3 which triggers Ca(2+) mobilization in eosinophils. [a.k.a. Q52LV8, NP_001358867.1, ENST00000005180, Eotaxin-3, eotaxin-3, ...]
EPO		Erythropoietin; Hormone involved in the regulation of erythrocyte proliferation and differentiation and the maintenance of a physiological level of circulating erythrocyte mass. Binds to EPOR leading to EPOR dimerization and JAK2 activation thereby activating specific downstream effectors, including
FLT3L		Fms-related tyrosine kinase 3 ligand; Stimulates the proliferation of early hematopoietic cells by activating FLT3. Synergizes well with a number of other colony stimulating factors and interleukins. [a.k.a. AAI36465.1, B9EGH2, XP_011524980.1, FLT3L, Flt3L, ...]
Fractalkine		Processed fractalkine; Acts as a ligand for both CX3CR1 and integrins. Binds to CX3CR1. Binds to integrins ITGAV:ITGB3 and ITGA4:ITGB1. Can activate integrins in both a CX3CR1-dependent and CX3CR1-independent manner. In the presence of CX3CR1, activates integrins by binding to the c
Gro-a		Growth-regulated alpha protein; Has chemotactic activity for neutrophils. May play a role in inflammation and exerts its effects on endothelial cells in an autocrine fashion. In vitro, the processed forms GRO-alpha(4-73), GRO- alpha(5-73) and GRO-alpha(6-73) show a 30-fold higher chemotactic activit
I-309	CCL1	C-C motif chemokine 1; Cytokine that is chemotactic for monocytes but not for neutrophils. Binds to CCR8; Belongs to the intercrine beta (chemokine CC) family. [a.k.a. R-HSA-388396, R-HSA-162582, NM_002981.2, I-309]
I-TAC	CXCL11	C-X-C motif chemokine 11; Chemotactic for interleukin-activated T-cells but not unstimulated T-cells, neutrophils or monocytes. Induces calcium release in activated T-cells. Binds to CXCR3. May play an important role in CNS diseases which involve T-cell recruitment. May play a role in skin immune
IFNb		Interferon beta; Has antiviral, antibacterial and anticancer activities; Belongs to the alpha/beta interferon family. [a.k.a. uc003zok.4, ENST00000380232, Interferon beta 1, IFNB]
IFNg		Interferon gamma; Produced by lymphocytes activated by specific antigens or mitogens. IFN-gamma, in addition to having antiviral activity, has important immunoregulatory functions. It is a potent activator of macrophages, it has antiproliferative effects on transformed cells and it can potentiate the ar
IL-10		Interleukin-10; Major immune regulatory cytokine that acts on many cells of the immune system where it has profound anti-inflammatory functions, limiting excessive tissue disruption caused by inflammation. Mechanistically, IL10 binds to its heterotetrameric receptor comprising IL10RA and IL10RB le
IL-12/IL-23p40		N/A
IL-13		Interleukin-13; Cytokine. Inhibits inflammatory cytokine production. Synergizes with IL2 in regulating interferon-gamma synthesis. May be critical in regulating inflammatory and immune responses. Positively regulates IL31RA expression in macrophages (By similarity). [a.k.a. ENST00000304506.7, N
IL-15		Interleukin-15; Cytokine that stimulates the proliferation of T-lymphocytes. Stimulation by IL15 requires interaction of IL15 with components of the IL2 receptor, including IL2RB and probably IL2RG but not IL2RA. In neutrophils, stimulates phagocytosis probably by signaling through the IL15 receptor, cc
IL-16		Pro-interleukin-16; Interleukin-16 stimulates a migratory response in CD4+ lymphocytes, monocytes, and eosinophils. Primes CD4+ T-cells for IL-2 and IL-15 responsiveness. Also induces T-lymphocyte expression of interleukin 2 receptor. Ligand for CD4. Isoform 3 is involved in cell cycle progressic
IL-17A/F		Interleukin-17A; Ligand for IL17RA and IL17RC. The heterodimer formed by IL17A and IL17F is a ligand for the heterodimeric complex formed by IL17RA and IL17RC. Involved in inducing stromal cells to produce proinflammatory and hematopoietic cytokines ; Belongs to the IL-17 family.Interleukin-17
IL-17B		Interleukin-17B; Stimulates the release of tumor necrosis factor alpha and IL- 1-beta from the monocytic cell line THP-1. [a.k.a. Interleukin-20, AF212311, AAK60336.1, IL-17B]
IL-17E/IL-25	IL25	Interleukin-25; Induces activation of NF-kappa-B and stimulates production of the proinflammatory chemokine IL-8. Proinflammatory cytokine favoring Th2-type immune responses; Belongs to the IL-17 family.
IL-17F		Interleukin-17F; Ligand for IL17RA and IL17RC. The heterodimer formed by IL17A and IL17F is a ligand for the heterodimeric complex formed by IL17RA and IL17RC. Involved in stimulating the production of other cytokines such as IL6, IL8 and CSF2, and in regulation of cartilage matrix turnover. Als
IL-18		Interleukin-18; A proinflammatory cytokine primarily involved in polarized T- helper 1 (Th1) cell and natural killer (NK) cell immune responses (Probable). Upon binding to IL18R1 and IL18RAP, forms a signaling ternary complex which activates NF-kappa-B, triggering synthesis of inflammatory mediat
IL-1Ra		Interleukin-1 receptor antagonist protein; Inhibits the activity of interleukin-1 by binding to receptor IL1R1 and preventing its association with the coreceptor IL1RAP for signaling. Has no interleukin-1 like activity. Binds functional interleukin-1 receptor IL1R1 with greater affinity than decoy receptor IL1R
IL-2		Interleukin-2; Produced by T-cells in response to antigenic or mitogenic stimulation, this protein is required for T-cell proliferation and other activities crucial to regulation of the immune response. Can stimulate B-cells, monocytes, lymphokine-activated killer cells, natural killer cells, and glioma cells. [a
IL-21		Interleukin-17C; Cytokine that plays a crucial role in innate immunity of the epithelium, including to intestinal bacterial pathogens, in an autocrine manner. Stimulates the production of antibacterial peptides and proinflammatory molecules for host defense by signaling through the NF-kappa-B and MAP
IL-22		Interleukin-22; Cytokine that contributes to the inflammatory response in vivo. [a.k.a. 1M4R, Q9GZX6, BC069308, IL-22]
IL-23		Interleukin-23 subunit alpha; Associates with IL12B to form the IL-23 interleukin, a heterodimeric cytokine which functions in innate and adaptive immunity. IL-23 may constitute with IL-17 an acute response to infection in peripheral tissues. IL-23 binds to a heterodimeric receptor complex composed c
IL-27		Myeloid-derived growth factor; Bone marrow-derived monocyte and paracrine-acting protein that promotes cardiac myocyte survival and adaptive angiogenesis for cardiac protection and/or repair after myocardial infarction (MI). Stimulates endothelial cell proliferation through a MAPK1/3-, STAT3- a
IL-29	IFNL1	Interferon lambda-1; Cytokine with antiviral, antitumour and immunomodulatory activities. Plays a critical role in the antiviral host defense, predominantly in the epithelial tissues. Acts as a ligand for the heterodimeric class II cytokine receptor composed of IL10RB and IFNLR1, and receptor engageme
IL-2Ra		Interleukin-2 receptor subunit alpha; Receptor for interleukin-2. The receptor is involved in the regulation of immune tolerance by controlling regulatory T cells (TREGs) activity. TREGs suppress the activation and expansion of autoreactive T-cells.
IL-3		Interleukin-3; Granulocyte/macrophage colony-stimulating factors are cytokines that act in hematopoiesis by controlling the production, differentiation, and function of 2 related white cell populations of the blood, the granulocytes and the monocytes-macrophages. [a.k.a. NP_000579.2, R-HSA-893924
IL-31		Interleukin-31; Activates STAT3 and possibly STAT1 and STAT5 through the IL31 heterodimeric receptor composed of IL31RA and OSMR. May function in skin immunity. Enhances myeloid progenitor cell survival in vitro (By similarity). Induces RETNLA and serum amyloid A protein expression in ma
IL-33		Interleukin-33 (109-270); Cytokine that binds to and signals through the IL1RL1/ST2 receptor which in turn activates NF-kappa-B and MAPK signaling pathways in target cells. Involved in the maturation of Th2 cells inducing the secretion of T-helper type 2-associated cytokines. Also involved in activat
IL-4		Interleukin-4; Participates in at least several B-cell activation processes as well as of other cell types. It is a costimulator of DNA-synthesis. It induces the expression of class II MHC molecules on resting B-cells. It enhances both secretion and cell surface expression of IgE and IgG1. It also regulates th
IL-5		Interleukin-5; Factor that induces terminal differentiation of late- developing B-cells to immunoglobulin secreting cells; Belongs to the IL-5 family. [a.k.a. R-HSA-912526, IL5-202, XM_011543373.2, IL-5]
IL-6		Interleukin-5; Factor that induces terminal differentiation of late- developing B-cells to immunoglobulin secreting cells; Belongs to the IL-5 family. [a.k.a. R-HSA-912526, IL5-202, XM_011543373.2, IL-5]
IL-7		Interleukin-7; Hematopoietic growth factor capable of stimulating the proliferation of lymphoid progenitors. It is important for proliferation during certain stages of B-cell maturation. [a.k.a. 3574, ENST00000639719.1, 3DI3, IL-7]
IL-8	CXCL8	Interleukin-8; IL-8 is a chemotactic factor that attracts neutrophils, basophils, and T-cells, but not monocytes. It is also involved in neutrophil activation. It is released from several cell types in response to an inflammatory stimulus. IL-8(6-77) has a 5-10-fold higher activity on neutrophil activation, IL-8(5-7
IL-9		Interleukin-9; Supports IL-2 independent and IL-4 independent growth of helper T-cells; Belongs to the IL-7/IL-9 family. [a.k.a. ENST00000274520.1, X17543, M30135, IL-9]
IP-10	CXCL10	C-X-C motif chemokine 10; Pro-inflammatory cytokine that is involved in a wide variety of processes such as chemotaxis, differentiation, and activation of peripheral immune cells, regulation of cell growth, apoptosis and modulation of angiostatic effects. Plays thereby an important role during viral infe
M-CSF		Processed macrophage colony-stimulating factor 1; Cytokine that plays an essential role in the regulation of survival, proliferation and differentiation of hematopoietic precursor cells, especially mononuclear phagocytes, such as macrophages and monocytes. Promotes the release of proinflammatory
MCP-1	CCL2	C-C motif chemokine 2; Acts as a ligand for C-C chemokine receptor CCR2. Signals through binding and activation of CCR2 and induces a strong chemotactic response and mobilization of intracellular calcium ions. Exhibits a chemotactic activity for monocytes and basophils but not neutrophils or e
MCP-2		C-C motif chemokine 8; Chemotactic factor that attracts monocytes, lymphocytes, basophils and eosinophils. May play a role in neoplasia and inflammatory host responses. This protein can bind heparin. The processed form MCP-2(6-76) does not show monocyte chemotactic activity, but inhibits the
MCP-4	CCL13	C-C motif chemokine 13, medium chain; Chemotactic factor that attracts monocytes, lymphocytes, basophils and eosinophils, but not neutrophils. Signals through CCR2B and CCR3 receptors. Plays a role in the accumulation of leukocytes at both sides of allergic and non-allergic inflammation. May
MDC	CCL22	Disintegrin and metalloproteinase domain-containing protein 11; Probable ligand for integrin in the brain. This is a non catalytic metalloprotease-like protein. [a.k.a. BAA04213.1, NP_001305862, ENSP00000200557, MDC]
MIF		Macrophage migration inhibitory factor; Pro-inflammatory cytokine. Involved in the innate immune response to bacterial pathogens. The expression of MIF at sites of inflammation suggests a role as mediator in regulating the function of macrophages in host defense. Counteracts the anti-inflammatory
MIP-1a	CCL3	C-C motif chemokine 3; Monokine with inflammatory and chemokinetic properties. Binds to CCR1, CCR4 and CCR5. One of the major HIV-suppressive factors produced by CD8+ T-cells. Recombinant MIP-1-alpha induces a dose- dependent inhibition of different strains of HIV-1, HIV-2, and simian
MIP-1b	CCL4	C-C motif chemokine 4; Monokine with inflammatory and chemokinetic properties. Binds to CCR5. One of the major HIV-suppressive factors produced by CD8+ T- cells. Recombinant MIP-1-beta induces a dose-dependent inhibition of different strains of HIV-1, HIV-2, and simian immunodeficiency v
MIP-3a	CCL20, LARC	C-C motif chemokine 20; Acts as a ligand for C-C chemokine receptor CCR6. Signals through binding and activation of CCR6 and induces a strong chemotactic response and mobilization of intracellular calcium ions. The ligand- receptor pair CCL20-CCR6 is responsible for the chemotaxis of denc
MIP-3b	CCL19, ELC	C-C motif chemokine 19; May play a role not only in inflammatory and immunological responses but also in normal lymphocyte recirculation and homing. May play an important role in trafficking of T-cells in thymus, and T-cell and B-cell migration to secondary lymphoid organs. Binds to chemokine rec
MIP-5	CCL15	C-C motif chemokine 15; Chemotactic factor that attracts T-cells and monocytes, but not neutrophils, eosinophils, or B-cells. Acts mainly via CC chemokine receptor CCR1. Also binds to CCR3. CCL15(22-92), CCL15(25-92) and CCL15(29-92) are more potent chemoattractants than the small-indu
SDF-1a		Stromal cell-derived factor 1; Chemoattractant active on T-lymphocytes and monocytes but not neutrophils. Activates the C-X-C chemokine receptor CXCR4 to induce a rapid and transient rise in the level of intracellular calcium ions and chemotaxis. SDF-1-beta(3-72) and SDF-1-alpha(3-67) show a
TARC		C-C motif chemokine 17; Chemotactic factor for T-lymphocytes but not monocytes or granulocytes. May play a role in T-cell development in thymus and in trafficking and activation of mature T-cells. Binds to CCR4. [a.k.a. D43767, 1NR4, C-C motif chemokine ligand 17, TARC]
TNFa		Tumor necrosis factor, membrane form; Cytokine that binds to TNFRSF1A/TNFR1 and TNFRSF1B/TNFBR. It is mainly secreted by macrophages and can induce cell death of certain tumor cell lines. It is potent pyrogen causing fever by direct action or by stimulation of interleukin-1 secretion and is ir
TPO	THPO, MGDF	Thrombopoietin; Lineage-specific cytokine affecting the proliferation and maturation of megakaryocytes from their committed progenitor cells. It acts at a late stage of megakaryocyte development. It may be the major physiological regulator of circulating platelets. [a.k.a. ENSG00000090534, ENST0
TRAIL	CD253, TNFSF	Tumor necrosis factor ligand superfamily member 10; Cytokine that binds to TNFRSF10A/TRAILR1, TNFRSF10B/TRAILR2, TNFRSF10C/TRAILR3, TNFRSF10D/TRAILR4 and possibly also to TNFRSF11B/OPG. Induces apoptosis. Its activity may be modulated by binding to the decoy receptors T
YKL-40		Chitinase-3-like protein 1; Carbohydrate-binding lectin with a preference for chitin. Has no chitinase activity. May play a role in tissue remodeling and in the capacity of cells to respond to and cope with changes in their environment. Plays a role in T-helper cell type 2 (Th2) inflammatory response and
VEGF-A	VEGF	N/A

Supplemental Table 4 | STRING description of each protein. Protein descriptions from STRING database.

medRxiv preprint doi: https://doi.org/10.1101/2024.04.22.24306188; this version posted June 11, 2024. The copyright holder for this preprint (which was not certified by peer review) is the author/funder, who has granted medRxiv a license to display the preprint in perpetuity.
This article is a US Government work. It is not subject to copyright under 17 USC 105 and is also made available for use under a CC0 license.

	True Negative	False Positive	False Negative	True Positive
**Logistic Regression with Class Weights**	121	19	2	3
**Logistic Regression with SMOTE**	114	26	1	4
**Random Forest with Class Weights**	137	3	4	1
**Gradient Boosting with Class Weights**	138	2	2	3
**XGBoost with Class Weights**	132	8	2	3

Supplemental Table 5 | Confusion Matrices for Models Applied to Determine Survival. Number of true negatives, false negatives, true positives, and false positives from a sample subset used to test the accuracy of machine learning models predicting death based on the expression levels of the 5-analyte panel.

medRxiv preprint doi: https://doi.org/10.1101/2024.04.22.24306188; this version posted June 11, 2024. The copyright holder for this preprint (which was not certified by peer review) is the author/funder, who has granted medRxiv a license to display the preprint in perpetuity. This article is a US Government work. It is not subject to copyright under 17 USC 105 and is also made available for use under a CC0 license.

SUPPLEMENTAL METHODS

Random Forest Model Tuning

Data was split 70/30 into training/test datasets, maintaining the ratio of trauma:healthy patients in each set. Hyperparameters were tuned with tidymodels grid search (n=20) to determine the appropriate number of variables to randomly sample at splitting (mtry) and minimum amount of data needed to split (min_n). Tuning was done with 10 trees using 5-fold cross validation to select the model with the highest AUC-ROC as the best model. This model was trained on the training set, then applied to test data. It was then validated on both training and test data separately with the same tuned mtry and min_n parameters and 200 trees. Predictions, model metrics, and variable permutation importance values from these final models were collected.

Random Forest Variable Importance

Permutation importance calculates the influence of each variable on model prediction based on how much a change in the variable's values affects the model's predictive error.[43] To derive summary statistics of variable importance values and to minimize the chance of a variable erroneously showing up as important, the model training and validation steps were repeated on the dataset 10 times from different seeds and the permutation importance for each variable was ranked for each iteration. The raw variable importance values were then converted to relative importance by dividing each variable importance score by the largest importance score of the variables for each of the 10 iterations and multiplied by 100.[54] Variables whose permutation importance ranked in the top 10 in at least 5 of the iterations were plotted, along with their minimum, first quartile, median, mean, third quartile, and maximum importance values.

SUPPLEMENTARY DISCUSSION

Confirmation of literature findings:

Our observation of decreased IL-2 levels, with many below the LOD, in trauma patients versus control samples is consistent with reports of reduced IL-2 production (associated with T cell activation) in response to traumatic injury.[64,65] In agreement with the literature, trauma patients also exhibited increased levels of several other interleukins, including IL-16,[66,67] IL-1Ra,[68,69] and

medRxiv preprint doi: https://doi.org/10.1101/2024.04.22.24306188; this version posted June 11, 2024. The copyright holder for this preprint (which was not certified by peer review) is the author/funder, who has granted medRxiv a license to display the preprint in perpetuity. This article is a US Government work. It is not subject to copyright under 17 USC 105 and is also made available for use under a CC0 license.

IL-6,[3] as well as a decrease in IL-21[4] and IL-3.[70] We observed several other patterns consistent with previous literature. These include MIF (upregulated[71]), TRAIL (upregulated and previously reported to be increased in elderly osteoporotic fractures[72]), and Eotaxin (downregulated, and previously reported to be downregulated in musculoskeletal surgical trauma[67,73]).

Downregulated proteins that are contrary to literature findings:

We also observed some trends that differ from those reported in the literature. The downregulation of VEGF-A, which is involved in angiogenesis, in trauma samples compared to healthy samples is especially interesting due to the involvement of angiogenesis in tissue repair. Although previous studies have demonstrated increased serum VEGF levels on day of arrival for both trauma and burn patients,[74] we observed a lower level of VEGF-A in trauma samples, regardless of injury mechanism, general wound type, specific wound type, wound location, or trauma level. This suggests a potential decrease in angiogenesis in the immediate aftermath of injury, possibly as a mechanism to control blood loss.

Of the other proteins for which we found trends that are contrary to what was previously published in the literature, evaluation of many of these proteins in human patients had primarily been limited to TBI. Increased levels of FLT3L, which activates FLT3 to stimulate proliferation of hematopoietic cells, have been reported in a study of the cerebral fluid of 10 patients that had experienced severe TBI.[20] In mice, FLT3L has been shown to exhibit protective effects in ischemia-reperfusion injury, volumetric muscle loss, and multi-organ dysfunction syndrome.[75–77] We observed significant decreases in FLT3L levels in trauma, as well as for each injury mechanism, general wound type, specific wound type, and wound location, versus healthy controls. This was also true for patients who had head/neck wounds, although it cannot be assumed that those patients would have TBI. Thus, the type of injury (ex. TBI vs. Non-TBI) likely played a role in the differences in our observation compared to the previous reports. In a study of brain tissue samples from 12 patients with severe TBI, I-TAC, which is chemotactic for IL-activated T-cells, was found to be upregulated. Although we observed an upregulation of I-TAC in trauma patients compared to healthy controls, this difference was not statistically significant. Nevertheless, other factors such as the use of systemic fluids (I.e. blood) versus localized fluids or tissues should also be taken into consideration, as it is possible that local elevation of proteins would not be reflected in systemically-sampled fluid.

medRxiv preprint doi: https://doi.org/10.1101/2024.04.22.24306188; this version posted June 11, 2024. The copyright holder for this preprint (which was not certified by peer review) is the author/funder, who has granted medRxiv a license to display the preprint in perpetuity. This article is a US Government work. It is not subject to copyright under 17 USC 105 and is also made available for use under a CC0 license.

Levels of IL-7, which is involved in stimulation of lymphoid progenitors, have previously been reported to be elevated with increasing injury severity for trauma patients,[4] although we observed a significantly lower level of IL-7 for trauma patients versus controls, and no significant difference between IL-7 levels in the most severe (level 1) cases compared to less severe (levels 2, 3, or 4) cases. However, this is likely a result of our inclusion of non-ICU patients, while the Cai et al. study included only trauma patients who were admitted to the ICU and survived to discharge. Another study found that TBI patients with chest injury had lower mean IL-7 levels than TBI patients without chest injury.[19] Although we observed a significant decrease in IL-7 levels across all wound types, wound locations, and injury mechanisms, these findings altogether suggest the importance of injury characteristics on IL-7 levels. This is further supported by conflicting reports on the levels of a number of other cytokines, which is likely a result of differences in patient characteristics. For example, IL-8 levels were shown to be higher in patients with orthopedic injures compared to healthy controls,[3] while serum IL-8 levels were shown to be higher in healthy controls compared to chest trauma patients.[78] Meanwhile, we observed lower IL-8 levels for trauma patients across all injury mechanisms, wound types, and wound locations, compared to healthy controls. TPO also has conflicting reports in the literature, with one study finding that patients with multiple trauma have elevated serum TPO in the days following injury,[79] while another study found that TPO levels were not measurable for patients with major lower extremity trauma.[80] Lower TPO levels have also been associated with nonsurvival in patients that have experienced major trauma.[81] We observed significantly lower TPO in trauma patients compared to healthy controls, regardless of injury mechanism, wound type, and wound location, However, patients with the most severe injuries exhibited higher TPO levels than patients with the least severe injuries, which appears to support prior findings of elevated TPO after severe injury.

A statistically significant decrease in concentration of IL-17E/IL-25 was observed for injury mechanisms of GSW and MVC but was not statistically significant for the other injury mechanisms. Additionally, the wound type played a role in the concentration of IL-17E/IL-25. Patients who experienced only internal wounds or only penetrating wounds did not exhibit a significant decrease in this protein, but those who experienced both wound types did. We observed that reduced IL-17E/IL-25 concentration was statistically significant for patients who experienced central wounds or combined head/neck and central wounds, but not for patients who experienced combined head/neck and peripheral wounds, head/neck wounds only, combined peripheral and central wounds, or peripheral wounds only.

medRxiv preprint doi: https://doi.org/10.1101/2024.04.22.24306188; this version posted June 11, 2024. The copyright holder for this preprint (which was not certified by peer review) is the author/funder, who has granted medRxiv a license to display the preprint in perpetuity. This article is a US Government work. It is not subject to copyright under 17 USC 105 and is also made available for use under a CC0 license.

Novel reporting of proteins with changes in levels below the LOD:

The concentration of several proteins (IL-31, IL-5, MIP-3a) previously unreported in this setting were not significantly associated with trauma; however, there were significant differences in our assay's ability to detect a signal. Each of these proteins were significantly less likely to be detected in trauma samples compared to healthy controls. This suggests an exceptional potential reduction in the concentrations of these proteins. IL-31 is linked both to Th2 immunity associated with wound healing and to dermatitis. IL-5 is also associated with type-2 immunity and eosinophil inflammation. MIP-3a has been associated with autoimmunity in mouse models, and also downregulated with IL-10 which correlates with trauma that induces IL-10. Type-2 immune proteins have important impact on wound closure and collagen deposition, which are critical in long-term recovery of injuries. Disruption of these pathways presents another mechanism by which severe trauma limits healing ability by decreasing the induction of collagen stimulating immune responses to close wounds and strength those closures. Induction of these responses locally may provide a therapeutic window to assist in wound healing that may be compromised in patients sustaining severe trauma.

Upregulated proteins that are contrary to literature findings:

While levels of both IL-22 and IL-23 have been reported to decrease in severe trauma,[4] we did not observe a significant change in IL-22 levels between trauma and healthy control patients. However, we found a significant increase in IL-23 concentration of trauma samples compared to healthy controls, which held true for patients injured through falls or stabbings, but not those who were injured in MVC or GSW. IL-23 levels are significantly higher in more severe (level 1) injuries, compared to less severe (level 3, level 4) injuries, and IL-22 levels were also significantly higher in level 1 versus level 4 injuries. Increased IL-23 levels were observed for patients who experienced only internal soft tissue wounds, or only penetrating soft tissue wounds, but not for patients with bone involvement. The observed differences between trends of IL-22 and IL-23 levels in our study compared to the previous report may be attributed to differences in patient cohorts, as the Cai et al. cohorts were comprised of only patients admitted to the ICU and who survived to discharge.
